# Monthly Increase in Vitamin D Levels upon Supplementation with 2000 IU/Day in Healthy Volunteers: Result from “Integriamoci”, a Pilot Pharmacokinetic Study

**DOI:** 10.3390/molecules27031042

**Published:** 2022-02-03

**Authors:** Valeria Avataneo, Alice Palermiti, Amedeo De Nicolò, Jessica Cusato, Gloria Giussani, Andrea Calcagno, Antonio D’Avolio

**Affiliations:** 1 Laboratory of Clinical Pharmacology and Pharmacogenetics, Department of Medical Sciences, Amedeo di Savoia Hospital, University of Turin, 10126 Turin, Italy; valeria.avataneo@unito.it (V.A.); alice.palermiti@unito.it (A.P.); jessica.cusato@unito.it (J.C.); antonio.davolio@unito.it (A.D.); 2 Witt Italia S.p.A., 10046 Poirino, Italy; g.giussani@witt.it; 3 Unit of Infectious Diseases, Department of Medical Sciences, University of Turin, 10124 Turin, Italy; andrea.calcagno@unito.it

**Keywords:** vitamin D, LC-MS/MS, oral integration, supplements, micronutrients

## Abstract

Vitamin D (VD) is a calcium- and phosphate-controlling hormone used to treat bone disorders; yet, several other effects are progressively emerging. VD deficiency is highly prevalent worldwide, with suboptimal exposure to sunlight listed among the leading causes: oral supplementation with either cholecalciferol or calcitriol is used. However, there is a scarcity of clinical studies investigating how quickly VD concentrations can increase after supplementation. In this pilot study, the commercial supplement ImmuD3 (by *Erboristeria Magentina*^®^) was chosen as the source of VD and 2000 IU/day was administered for one month to 21 healthy volunteers that had not taken any other VD supplements in the previous 30 days. Plasma VD levels were measured through liquid chromatography coupled to tandem mass spectrometry after 7, 14, and 28 days of supplementation. We found that 95% of the participants had insufficient VD levels at baseline (<30 ng/mL; median 23.72 ng/mL; IQR 18.10–26.15), but after 28 days of supplementation, this percentage dropped to 62% (median 28.35 ng/mL; IQR 25.78–35.20). The median increase in VD level was 3.09 ng/mL (IQR 1.60–5.68) after 7 days and 8.85 ng/mL (IQR 2.85–13.97F) after 28 days. This study suggests the need for continuing VD supplementation and for measuring target level attainment.

## 1. Introduction

Vitamin D (VD) is historically a critical nutrient for rickets prevention and treatment. This nutrient is considered essential for bone and mineral metabolism and thus for musculoskeletal system wellness. Furthermore, supporting a broader role of VD in human health with key endocrine functions, associations between low VD levels and various extra-skeletal diseases such as cancer [1,2,3,4,5], infections [6,7,8,9,10,11,12,13], and some cardiovascular diseases [14] have also been reported. Moreover, the cut-off levels considered as “VD deficiency” and “VD insufficiency” have been widely variable in the last years, mainly based on various healthcare contexts [15,16,17,18,19,20]. In the context of bone metabolism, VD levels (especially 25-hydroxyvitamin D (25(OH)D)) lower than 30 nmol/L (corresponding to 12 ng/mL) can cause osteomalacia and rickets; no further benefits seem to be observed with levels higher than 50 nmol/L (20 ng/mL) [16]. Therefore, the US Institute of Medicine (IOM), as well as the Endocrine Society, recommended a daily allowance (RDA) for dietary VD intake between 400 and 800 international units (IU) per day, depending on patients’ age, as summarized in Table 1 [16,18].

Dietary intake can only contribute up to 20% of the total VD requirement. There is scientific controversy over the extra-skeletal effects of VD, which are associated with higher 25(OH)VD levels than those that can be considered sufficient for calcium and phosphorous homeostasis. Additionally, due to the evidence of widespread worldwide VD deficiency (often dependent on poor exposure to sunlight for health, geographical, ethnic reasons, etc.), several studies and guidelines began suggesting an increase in the traditional intake levels, as needed [18,21]. In particular, the clinical practice guidelines by the US Endocrine Society [18] stated how a significantly higher daily intake (1500–2000 IU in adults) would be needed in order to reach a target VD level of 30 ng/mL, which was considered associated with further skeletal and extra-skeletal benefits in several studies [21,22,23]. These higher suggested RDAs are acceptable within the upper daily allowance suggested both by the IOM and the European Food Safety Authority (EFSA), summarized in Table 2, which are as high as 4000 IU/day in healthy adults [16,17,24]. Moreover, several reports highlighted that cases of VD-related toxicity were only observed at extremely high doses and concentrations (more than 40,000 IU/day for several days and over 150 ng/mL, respectively), indicating that the upper daily allowances defined by the IOM and EFSA provide a considerable margin of safety [18,25].

In recent years, particularly in light of the COVID-19 pandemic, VD deserved attention for its effects on the immune system: VD can act as an immunomodulator of innate and adaptive immune responses. Functional VD receptors (VDRs) have been identified in nearly all immune cells, including activated CD4+ and CD8+ T cells, B cells, neutrophils, and antigen-presenting cells (APCs), such as macrophages and dendritic cells (DCs), as well as in human airway epithelial cells [6]. Furthermore, in vitro studies have shown that VD decreases the expression of pro-inflammatory cytokines and increases the production of antiviral proteins, suggesting an essential role in innate antiviral immunity and involvement in inflammatory processes. In humans, cross-sectional clinical studies have shown that lower serum VD levels are significantly associated with respiratory tract infections, including epidemic influenza and tuberculosis (TB) [8,10,11,26,27]. For example, a British cohort study revealed that the prevalence of respiratory infections showed a strong seasonal pattern in the opposite direction to that of serum 25(OH)D concentrations [7]. In the context of TB, some authors evidenced the in vitro anti-mycobacterial activity induced by calcitriol, through the modulation of the host response, to mycobacterial infection. VD is also responsible for cathelicidin secretion, which stimulates the autophagy of *M. tuberculosis* [28,29,30,31]. Furthermore, low VD concentrations in serum were associated with a higher risk of developing multidrug-resistant TB [32]. A meta-analysis of randomized controlled trials reported that VD supplementation could reduce the risk of experiencing at least one acute respiratory tract infection. Furthermore, a subgroup analysis revealed that daily or weekly VD intake protected against acute respiratory tract infection, whereas regimens containing large bolus doses did not. A proposed explanation speculates that high circulating concentrations after bolus dosing may chronically deregulate the activity of enzymes responsible for synthesis and degradation of the active VD metabolite, resulting in decreased concentrations of this metabolite in extra-renal tissues [12]. Regarding the optimal serum 25(OH)D levels, a prospective cohort study conducted on 198 healthy adults showed that concentrations ≥100 nmol/L (40 ng/mL) could provide strong protection against acute viral respiratory infections [13]. Nevertheless, to reach these concentrations in adults, a VD supplementation of up to 4000–6000 IU/day (depending on the baseline level), still considered safe, would be required [18,21]. Last but not least, several researchers proposed VD deficiency or insufficiency as a risk factor for SARS-CoV-2 infection and the worst clinical evolution for COVID-19 illness [33,34]. In this same context, other works reported better survival rates in COVID-19 patients with VD levels over 30 ng/mL [35,36], paving the way to clinical trials involving high bolus supplementation protocols in infected patients [37,38]. According to the study by Annweiler et al., patients who received a constant supplementation and had higher VD levels at baseline had higher survival rates than patients treated with 25(OH)VD bolus and untreated patients [37]. Therefore, a preventive correction of VD deficiency or insufficiency, reaching values higher than 30 ng/mL, seems a safe and effective measure to improve general health status and to mitigate the impact of COVID-19. The evidence indicates that doses higher than 1500 IU/day would be needed to reach this threshold. For this reason, in this study, we aimed to describe the effectiveness of a one-month supplementation with a commercial VD supplement at 2000 IU/day (well within the upper daily allowance values suggested by the IOM and EFSA) in reaching a VD cutoff value of 30 ng/mL in a cohort of healthy adult volunteers.

## 2. Materials and Methods

### 2.1. Study Design and Participants Enrolment

Healthy volunteers, aged between 18 and 75 years old, were enrolled in the Italian clinical study “Integriamoci: self-microsampling evaluation of vitamin D3 plasma concentrations following the intake of ImmuD3 (Erboristeria Magentina)” (approved by the Bioethics Committee of the University of Turin and by the ASL “Città di Torino”, study protocol: 532/02.06/2021, 17 May 2021). Inclusion criteria entailed being an adult, without any recent (last 6 months) history of VD supplementation and having a BMI in the range of 20 to 25 kg/m^2^. The presence of known diseases (particularly involving calcium and phosphorous homeostasis, musculoskeletal diseases, hyperparathyroidism, hypertension, kidney or hepatic disease, and obesity) were considered exclusion criteria. After signing the informed consent form, each participant received a sample of ImmuD3 with the recommendation to take 2000 IU/day, corresponding to two drops. Patient’s compliance was monitored through a questionnaire that was checked daily after the intake. Capillary blood sampling was performed upon the signing of informed consent at the Laboratory of Clinical Pharmacology and Pharmacogenetics of Amedeo di Savoia Hospital of Turin and collected at baseline (T0) and 7 (T7), 14 (T14), and 28 (T28) days after supplementation began. The study was conducted between May and June 2021.

### 2.2. Vitamin D Measurement

Samples were autonomously collected after assisted finger-pricking, using Microvette^®^ 200 Lithium heparin tubes (Sarstedt^®^, Numbrecht, Germany). Each blood sample was centrifuged at room temperature for 5 min at 2000× *g*, to obtain plasma, and subsequently stored at −20°C. We measured 25(OH)D levels by using an MSMS Vitamin D kit (Perkin Elmer^®^, Milan, Italy). Extraction was performed according to the protocol. A Perkin Elmer LX-50^®^ UHPLC system coupled with a Triple Quadrupole QSight 220^®^ (Perkin Elmer, Milan, Italy) was used for the chromatographic analysis. The method performance was evaluated in terms of accuracy, repeatability, and reproducibility on 5 intra-day replicates and in 6 different inter-day sessions, yielding a mean accuracy of 98.6%, intra-day coefficient of variation (CV) of 3.5%, and inter-day CV of 8.9%.

The method was also evaluated for mutual concordance with the IVD COBAS VD platform by Passing-Bablok regression, showing an excellent concordance (concentration by LC-MS/MS = 1.063 × (concentration by COBAS) − 0.903). The method comparison results are reported in Figures S1 and S2 (Passing-Bablok regression and Altman-Bland plot).

Per SACN guidelines, the VD concentration groups were assigned as follows: VD deficiency as 25(OH)D < 20 ng/mL, insufficiency as 20–30 ng/mL, and sufficiency as >30 ng/mL [19].

### 2.3. Statistical Analysis

Statistical analyses were performed using IBM SPSS software, version 27.0. Due to the non-normal distribution of paired data, the differences between blood-collection timings were tested by the nonparametric Wilcoxon test (for two groups). Bivariate correlations were studied using the Spearman test such as those between VD levels and age or ultraviolet (UV) light exposure (self-reported by patients, described as follow: 0 = <½ h/day; 1 = ½–1 h/day; 2 = 1–3 h/day; 3 = >3 h/day).

## 3. Results

Twenty-one Caucasic healthy volunteers were enrolled in the study; of them, 12 were women. The median age was 45 years (interquartile range (IQR): 35–53). One person declared a UV exposure of less than half an hour per day, eight subjects reported half to one hour per day, while the remaining twelve reported one to three hours per day. None reported a UV exposure higher than 3 h/day. The volunteers’ characteristics at baseline are reported in Table 3.

**Figure 1:**
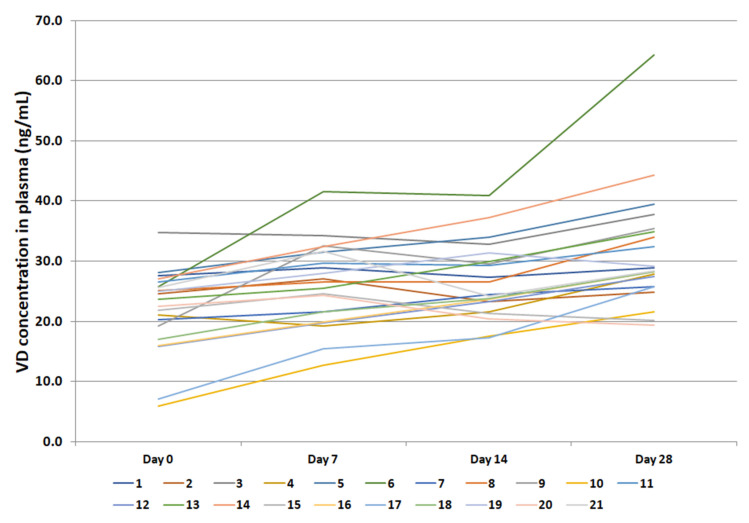
Overview of patient’s results in terms of monthly vitamin D (VD) increase following oral supplementation with 2000 IU/day.

**Figure 2:**
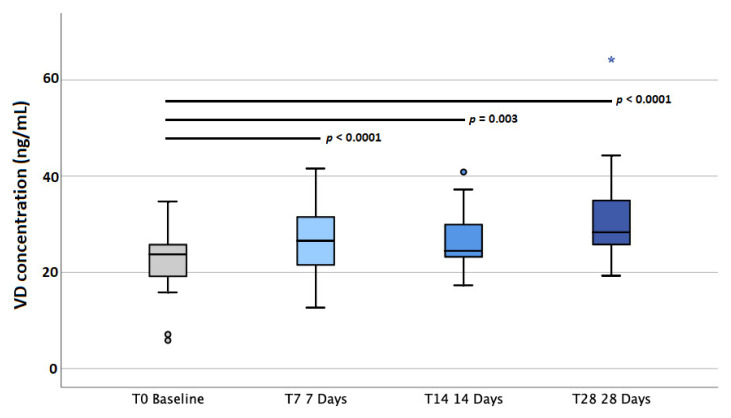
Plasma vitamin D (VD) concentrations measured at baseline and at 7, 14, and 28 days after the beginning of supplementation.

**Figure 3:**
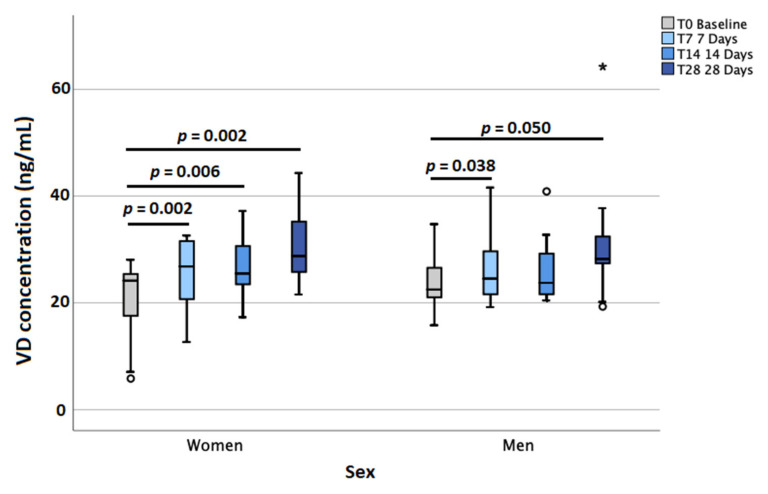
Plasma vitamin D (VD) monthly increase according to sex. No correlations were found between VD increases and age or UV exposure, respectively.

At baseline, 95% of participants showed insufficient VD levels (<30 ng/mL) and 29% displayed deficient (<20 ng/mL) levels; after 28 days, 62% still possessed insufficient concentrations but only 5% were deficient (Figure 1 and Supplementary Table S1). Median VD concentrations were as follows: 23.72 ng/mL (IQR 18.10–26.15) at baseline, 26.55 ng/mL (IQR 20.69–31.55) at T7, 24.46 ng/mL (IQR 22.41–30.62) at T14, and 28.35 ng/mL (IQR 25.78–35.20) at T28. By dividing according to sex, women achieved the following results: 24.15 ng/mL (IQR 16.78–25.52) at baseline, 26.81 ng/mL (IQR 20.26–31.57) at T7, 25.49 ng/mL (IQR 23.34–30.96) at T14, and 28.76 ng/mL (IQR 25.77–35.34) at T28. For men, the results were as follows: 22.49 ng/mL (IQR 19.03–27.09) at baseline, 24.52 ng/mL (IQR 20.68–31.97) at T7, 23.73 ng/mL (IQR 21.44–30.98) at T14, and 28.22 ng/mL (IQR 23.78–35.06) at T28. The Wilcoxon test revealed that baseline VD levels were significantly different from those obtained at T7 (*p* < 0.0001), T14 (*p* = 0.003) and T28 (*p* < 0.0001) (Figure 2). By dividing according to sex, the significance was confirmed at all three timings for women and T7 and T28 for men (Figure 3). The median VD level increase was 3.09 ng/mL (IQR 1.60–5.68) at T7, 5.87 ng/mL (IQR −0.44–8.95) at T14, and 8.85 (IQR 2.85–13.97) ng/mL at T28. By grouping according to sex, women showed higher increases: 3.64 ng/mL (IQR 1.99–6.62) at T7, 6.28 ng/mL (IQR 2.09–10.19 at T14, and 11.33 ng/mL (IQR 4.54–16.16) at T28. On the other hand, men achieved the following increases: 2.63 ng/mL (IQR 0.41–4.26) at T7, 0.57 ng/mL (IQR −1.29–7.07) at T14, and 5.85 ng/mL (IQR −0.20–11.39) at T28.

## 4. Discussion

This study aimed to describe VD through concentrations upon consumption of a commercially available nutritional supplement at a dosage of 2000 IU/day, which is emerging as better than the widely recommended 800 IU, particularly when employed for immunomodulatory purposes, aiming at levels higher than 30 ng/mL. This is important in light of the recent interest in the context of the COVID-19 pandemic. For these reasons, the most stringent level of 30 ng/mL was chosen, per SACN guidelines and a vast body of research reporting significantly lower risk of SARS-CoV-2 infection and COVID-19 severity for patients over this cut-off value [19,33,34,35,36].

Interestingly, in this study the absence of correlations between VD increases and UV exposure indicated that the observed results are mainly attributable to supplementation and not to possible sun exposure. The results demonstrated that 95% of the volunteers showed insufficient VD levels at baseline, lower than 30 ng/mL, confirming the high prevalence of VD insufficiency at the end of winter, even in southern Europe, adding to the strong rationale for VD supplementation in this context. On the other hand, after 28 days, this percentage fell to 62%, remaining consistent. This is in accordance with other studies that reported a mean time of 68.4 days to reach a VD level >30 ng/mL by assuming 800 IU/day [39]. In this case, we would expect a shorter period, but consistently longer than one month. On the other hand, 29% of the participants were deficient in VD (values lower than 20 ng/mL) at baseline but after only 28 days of supplementation, this percentage fell to 5% (1 subject). This confirms the effectiveness of a 2000 IU/day supplementation regimen to rapidly correct VD deficiency. These are important results, suggesting the need to continue supplementation for longer than one month or adopt a higher VD dosage (e.g., 4000 UI/day) when aiming for immune benefits, particularly in men. The dosage adopted in this study seems to be more suitable than the 800 IU/day, in accordance with the clinical practice guidelines of the Endocrine Society or the European Vitamin D Association (EVIDAS) guidelines [18,20], which already suggested VD supplementation with 2000 IU/day in adults during periods of minimum sunlight exposure. We observed a median approximate increase of 9 ng/mL in the course of 30 days, which is slightly higher than observed in a study by Smith et al. in the context of a study conducted during winter in Antarctica, which had the great advantage of the total absence of confounding factors, but it was also extremely far from a real-life context, such as in the present study [40]. A considerable inter-subject variability in the increase in VD levels during the protocol was observed in our cohort, which may be explained by inter-patient differences in body weight, BMI, sex, and genetics (notably, polymorphisms on genes involved in its activation and inactivation such as CYP2R1, CYP27B1, and CYP24A1) [41,42]. Unfortunately, studying the effect of differences in anthropometric variables on VD concentrations was not a primary endpoint of this study. These data were not possible to collect for ethical reasons: in order to limit the impact of major differences in BMI, only volunteers in a range between 20 and 25 kg/m^2^ were enrolled. Moreover, it is important to note that major confounding factors for VD pharmacokinetics and disposition, such as hepatic or renal diseases and obesity, were part of the exclusion criteria in this study. Concerning sex stratification, a slightly different trend was observed among men and women, with the latter characterized by a more sudden increase. Although an obvious explanation cannot be deduced from this study, these differences could be explained by the discrepancies in body weight and BMI between male and female participants, adding an interesting topic for a future study.

One of the major limitations of this study is the absence of a control group; but, in the context of COVID-19 pandemic, with the emerging evidence of an important role of VD in terms of symptoms severity reduction, it has been difficult to find healthy volunteers not taking supplementation and without the willpower to begin it.

## 5. Conclusions

In this study, the evidence of a wide prevalence of VD insufficiency at baseline strongly supports the rationale for VD supplementation and monitoring. The supplementation with 2000 IU/day of VD_3_ appeared partially effective at increasing the overall 25(OH)VD_3_ levels above a cut-off value of 30 ng/mL, suggested as optimal for preventing respiratory infectious disease. Nevertheless, the high proportion of volunteers that had VD concentrations still under 30 ng/mL at the end of the protocol suggests that a longer supplementation period or with higher VD doses, such as 4000 UI/day, would be needed to increase its effectiveness when aiming at immune benefits. This point, as well as the impact of body weight and BMI on VD concentrations, deserve further investigation in the near future.

## Figures and Tables

**Table 1:** RDA needed to reach vitamin D target levels in different age considering IOM and Endocrine Society Clinical Practice guidelines [16,18].

		IOM	Endocrine Society		
Age	Target	RDA	RDA	RDA to Reach High Target	High Target
<1 years	>12 ng/mL	400 IU/day 600 IU/day 600 IU/day 600 IU/day 800 IU/day	400 IU/day	-	-
1–18 years	>20 ng/mL		400–600 IU/day	>1000 IU/day	30 ng/mL
19–50 years	>20 ng/mL		>600 IU/day	1500–2000 IU/day	30 ng/mL
50–70 years	>20 ng/mL		>600 IU/day	1500–2000 IU/day	30 ng/mL
>70 years	>20 ng/mL		800 IU/day	1500–2000 IU/day	30 ng/mL

**Table 2:** Upper limits of vitamin D intake at different ages considering the IOM and EFSA guidelines [16,17].

Upper Limits for Vitamin D Intake		
Age	IOM	EFSA
<6 months	1000 IU/day	1000 IU/day
>6 months	1500 IU/day	1000 IU/day
1–3 years	2500 IU/day	2000 IU/day
4–8 years	3000 IU/day	2000 IU/day
9–10 years	4000 IU/day	2000 IU/day
>11 years	4000 IU/day	4000 IU/day

**Table 3:** Volunteers’ characteristics at the baseline.

Number of volunteers	21
Age (Median, IQR)	45 (37–52)
Sex (% male)	42.8%
BMI (range)	20–25
UV exposure <½ h/day	1 (4.76%)
½–1 h/day	10 (47.62%)
1–3 h/day	14 (66.67%)
>3 h/day	0
Median VD level at baseline (ng/mL, IQR)	23.72 (18.10–26.15)

## Data Availability

Raw data will be provided upon request.

## Supplementary Materials

Supplementary Materials are available online. Supplementary Figure S1: Passing-Bablok regression plot for the comparison between the LC-MS/MS and COBAS IVD immunoassay; Figure S2: Altman–Bland plot of the residuals in the comparison between LC-MS/MS and COBAS IVD immunoassay; Table S1: Detailed description of VD concentrations during the supplementation protocol in all the enrolled volunteers.

## Sample Availability

Not available.

## Abbreviations

VD: vitamin D; IU, international units; IQR, inter-quartile range; 25(OH)D, 25-hydroxyvitamin D; RDA, recommended daily allowance; EFSA, European Food Safety Authority; IOM, Institute of Medicine; VDRs, vitamin D receptors; DCs, dendritic cells; TB, tuberculosis; T0, baseline; T7, seven days; T14, fourteen days; T28, twenty-eight days; UV, ultraviolet.
