# Supplementary material for: Monthly Increase in Vitamin D Levels upon Supplementation with 2000 IU/Day in Healthy Volunteers: Result from “Integriamoci”, a Pilot Pharmacokinetic Study
Source: Molecules. 2022 Feb 3;27(3):1042. doi: 10.3390/molecules27031042 (PMC8840528; doi:10.3390/molecules27031042)
Supplement: Supplementary file 1 [file molecules-27-01042-s001.zip › molecules-1548158-supplementary/Supplementary Figures S1 and S2.pdf]

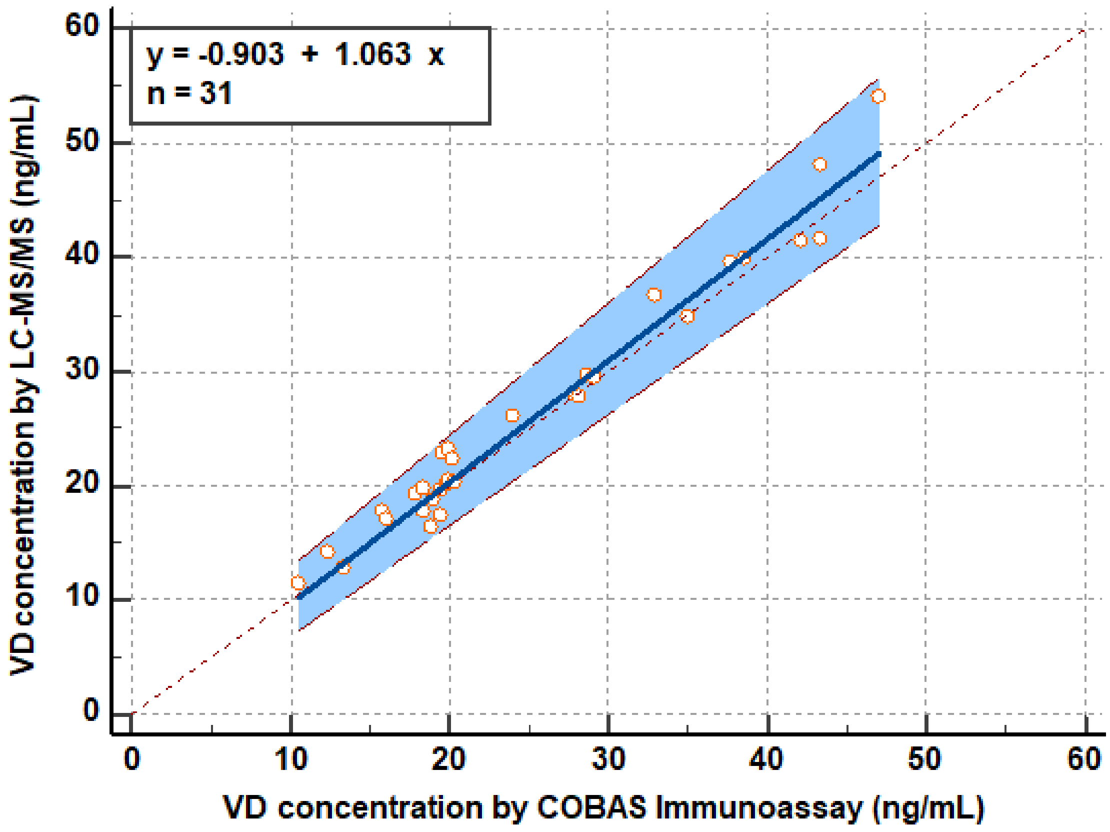

Supplementary figure s1: Passing-Bablok regression. The dashed lines represent the 95% confidence interval for the regression curve.

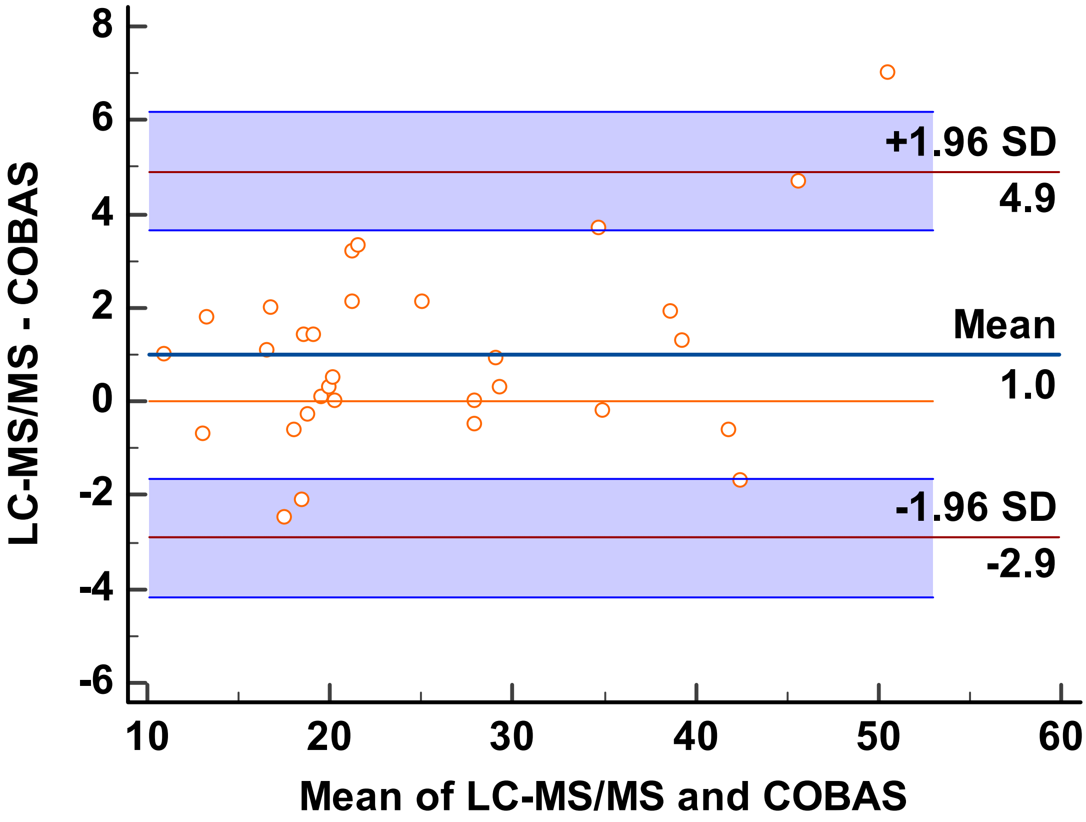

Supplementary Figure s2: Altman-Bland plot of residuals for the comparison between analytical results by LC-MS/MS and COBAS immunoassay.
