# Supplementary material for: Monthly Increase in Vitamin D Levels upon Supplementation with 2000 IU/Day in Healthy Volunteers: Result from “Integriamoci”, a Pilot Pharmacokinetic Study
Source: Molecules. 2022 Feb 3;27(3):1042. doi: 10.3390/molecules27031042 (PMC8840528; doi:10.3390/molecules27031042)
Supplement: Supplementary file 1 [file molecules-27-01042-s001.zip › molecules-1548158-supplementary/Supplementary Table 1.pdf]

	VD concentrations in plasma (ng/mL)			
Volunteer ID	Baseline (day 0)	Day 7	Day 14	Day 28
1	27.6	28.9	27.4	29.0
2	24.6	27.1	23.2	24.8
3	34.7	34.3	32.7	37.7
4	21.0	19.2	21.6	27.8
5	28.1	31.5	33.9	39.5
6	25.7	41.6	40.9	64.2
7	20.3	21.5	24.5	25.8
8	25.1	26.5	26.5	34.0
9	19.2	32.6	29.5	35.5
10	5.8	12.7	17.5	21.6
11	26.6	29.6	29.2	32.4
12	15.8	19.8	23.3	27.4
13	23.7	25.5	29.9	34.9
14	27.0	32.4	37.2	44.3
15	21.9	24.5	21.3	20.2
16	16.0	19.8	23.7	28.2
17	7.1	15.4	17.3	25.8
18	17.0	21.6	23.7	28.2
19	25.0	27.9	31.3	29.2
20	22.5	24.3	20.4	19.3
21	25.6	31.6	24.2	28.3

Supplementary table S1: detailed description of VD concentrations during the supplementation protocol in all the enrolled volunteers
